# The Biological Role of Hyaluronan-Rich Oocyte-Cumulus Extracellular Matrix in Female Reproduction

**DOI:** 10.3390/ijms19010283

**Published:** 2018-01-18

**Authors:** Eva Nagyova

**Affiliations:** Institute of Animal Physiology and Genetics, Academy of Sciences of the Czech Republic, 27721 Libechov, Czech Republic; nagyova@iapg.cas.cz; Tel.: +420-315-639564

**Keywords:** extracellular matrix, hyaluronan, inter-alpha-trypsin inhibitor, tumor necrosis factor-alpha-induced protein 6, pentraxin 3, oocyte-cumulus complexes

## Abstract

Fertilization of the mammalian oocyte requires interactions between spermatozoa and expanded cumulus extracellular matrix (ECM) that surrounds the oocyte. This review focuses on key molecules that play an important role in the formation of the cumulus ECM, generated by the oocyte-cumulus complex. In particular, the specific inhibitors (AG1478, lapatinib, indomethacin and MG132) and progesterone receptor antagonist (RU486) exerting their effects through the remodeling of the ECM of the cumulus cells surrounding the oocyte have been described. After gonadotropin stimulus, cumulus cells expand and form hyaluronan (HA)-rich cumulus ECM. In pigs, the proper structure of the cumulus ECM depends on the interaction between HA and serum-derived proteins of the inter-alpha-trypsin inhibitor (IαI) protein family. We have demonstrated the synthesis of HA by cumulus cells, and the presence of the IαI, tumor necrosis factor-alpha-induced protein 6 and pentraxin 3 in expanding oocyte-cumulus complexes (OCC). We have evaluated the covalent linkage of heavy chains of IαI proteins to HA, as the principal component of the expanded HA-rich cumulus ECM, in porcine OCC cultured in medium with specific inhibitors: AG1478 and lapatinib (both inhibitors of epidermal growth factor receptor tyrosine kinase activity); MG132 (a specific proteasomal inhibitor), indomethacin (cyclooxygenase inhibitor); and progesterone receptor antagonist (RU486). We have found that both RU486 and indomethacin does not disrupt the formation of the covalent linkage between the heavy chains of IαI to HA in the expanded OCC. In contrast, the inhibitors AG1478 and lapatinib prevent gonadotropin-induced cumulus expansion. Finally, the formation of oocyte-cumulus ECM relying on the covalent transfer of heavy chains of IαI molecules to HA has been inhibited in the presence of MG132.

## 1. Extracellular Matrix in General

The extracellular matrix (ECM) is an important structure that is present in all tissues. The ECM interacts with cells to regulate a wide range of functions, including adhesion, proliferation, apoptosis and differentiation. The ECM can also locally release growth factors, such as epidermal growth factor (EGF), fibroblast growth factor and other signaling molecules such as transforming growth factor (TGFβ) and amphiregulin [1]. Naba et al. [2] defined ECM proteins of the mammalian matrisome by analysis of the human and mouse genome. It comprises 1–1.5% of the mammalian proteome. There are almost 300 proteins, including 43 collagen subunits, 36 proteoglycans (e.g., aggrecan, versican, perlecan and decorin) and ~200 complex glycoproteins (e.g., laminins, elastin, fibronectins, thrombospondins, tenascins or nidogen). Moreover, there are large numbers of ECM-modifying enzymes, ECM-binding growth factors, and other ECM-associated proteins [3]. Proteoglycans are important structural macromolecules in tissues. They consist of a core protein with attached glycosaminoglycan side chains. There are six types of glycosaminoglycans: chondroitin sulfate, dermatan sulfate, heparin sulfate, heparin, keratin sulfate and hyaluronan (HA). Hyaluronan is the only glycosaminoglycan synthesized at the cell membrane and it is present in a protein-free form [4]. The size of HA depends on the relative activity of HA-synthesizing and degrading enzymes. There are three hyaluronan synthase isoforms (HAS1, 2 and 3) [5]. The expression of the three HAS isoforms is regulated by various stimuli, suggesting different functions of the three proteins [6]. In contrast, hyaluronidases 1–4 degrade HA into several fragments with size-dependent functions [7]. Dysregulation of ECM structure causes tissue malfunction such as inflammation, infertility and cancer. The importance of the function of the ECM is demonstrated by the embryonic lethality caused by mutations in genes that encode components of the ECM [8,9].

## 2. Cumulus–Oophorus Extracellular Matrix in Ovarian Follicles: Characterization of Essential Components

It has been shown that the complex of heavy chains of inter-alpha-trypsin-inhibitor (IαI) to HA is the principal structural component of the cumulus ECM in ovarian follicles in mice [10,11] and pigs [12]. Genetic deletion of specific ECM proteins such as bikunin (light chain of IαI) and tumor necrosis factor alpha-induced protein 6 (*Tnfaip6-*null mice were unable to transfer heavy chains from IαI to HA) exhibit infertility in female mice [11,13]; see Table 1. Suzuki et al. [14] identified the full repertoire of the IαI deficiency-related genes from bikunin-knockout female mice. They suggested that proteins of the IαI family have additional effects on reproductive biology by modulating the expression of a large number of cellular genes.

### 2.1. IαI Family Proteins

It has been demonstrated that mice lacking intact IαI family proteins fail to form a stable cumulus ECM, and the naked ovulated oocytes are not fertilized in vivo [11]. Importantly, HA-rich oocyte-cumulus ECM does not form in IαI immune-depleted serum, while it does in the presence of purified IαI molecules [20]. The proper structure of the cumulus ECM depends on the interaction between HA and serum-derived proteins of the IαI family [11,12,21]. The IαI family proteins are synthesized and assembled in the liver and secreted into the blood at high concentrations (0.15–0.5 mg/mL of plasma) [35]. These IαI molecules consist of a small protein, named bikunin or light chain, with a chondroitin sulfate moiety that contains one or two evolutionarily related proteins, named heavy chains (HC1, HC2, and HC3). The first member, IαI, carries two heavy chains, HC1 and HC2, while the next two members, pre-α-inhibitor (PαI) and inter-α-like inhibitor (IαLI) have one heavy chain, i.e., HC, HC3 and HC2, respectively [36]. Positive bands of about 220, 130, and 120 kDa were detected in porcine serum [12] that likely correspond to IαI (bikunin plus HC1 and HC2), PαI (bikunin plus HC3), and IαLI (bikunin plus HC2), respectively [37,38]. In addition, porcine follicular fluids, collected at different stages of folliculogenesis were analyzed for the presence of IαI proteins [12]. Importantly, the levels of IαI molecules in porcine follicular fluid did not change in PMSG-primed follicles or in 8 h hCG-stimulated follicles, while a detectable increase in concentration was observed at 24 h post-hCG injection. In pigs, there is no apparent barrier to the transfer of IαI family molecules from the blood to the follicles, and the LH/hCG stimulation only facilitates their diffusion. Inside the follicle, the heavy chains are transferred from the glycosaminoglycan of IαI-related molecules to HA through a transesterification process [21]. To determine, whether HCs of serum-derived IαI-related molecules are covalently linked to HA in porcine expanded OCC in vivo, the experiments with OCC isolated from the antral follicles of pigs treated with PMSG (unexpanded OCC) or PMSG followed by hCG for 24 h (expanded OCC) were performed [12]. The authors found that expanded OCC contained positive bands of about 220, 130, and 120 kDa that likely correspond to IαI, PαI and IαLI, respectively [12,37,38]. After digestion of the expanded complexes with hyaluronidase, two additional immunopositive bands of about 85 kDa and 95 kDa were detected in the matrix extracts, the former likely corresponding to the relative molecular mass of single HC1 and HC2, and the latter to that of a single HC3 [36,39]. In addition, IαI proteins were detected in porcine OCC expanded in vitro after their culture in FSH- and serum-supplemented medium [12]. Interestingly, it has been shown that HCs from each IαI-related molecule identified in the serum are transferred and covalently linked to HA during the cumulus expansion of OCC. Analysis of the cumulus matrix and cell extracts clearly confirmed that the immunoreactivity was associated with the HA-rich cumulus ECM [12]. In mice and pigs [12,15,21], it has been confirmed that the covalent linkage of the heavy chains of IαI to HA is critical for cross-linking HA strands and stabilizing expanded HA-rich cumulus ECM. 

### 2.2. TNFAIP6

It has been shown that the covalent transfer of heavy chains (HCs) of IαI proteins to HA does not occur in the OCC of *Tnfaip6*-null mice, indicating that TNFAIP6 is actively involved in this process [13]. TNFAIP6 is an inflammation-associated protein with the ability to bind HA, IαI, and other ligands and participate in the cumulus ECM formation and remodeling [40,41,42]. TNFAIP6 is produced by cumulus and granulosa cells after an ovulatory stimulus in mice, rats and pigs [13,18,24,25,26,27,28]. Previously, in pigs, four bands were detected with the antibody specific for TNFAIP6 in total and matrix protein extracts from OCC expanded in vivo. The major positive band had an apparent molecular weight of 35 kDa that also correlated well with the size of the free TNFAIP6 protein in mouse OCC. A doublet at ~120 kDa (HC-TNFAIP6 complex in mouse and pig) was also immunoreactive with the anti-TNFAIP6 antibody. In addition, the TNFAIP6 protein was detected in porcine OCC expanded in vitro after their culture in FSH- and serum-supplemented medium for 24 and 42 h [26]. It has been demonstrated that TNFAIP6, which binds to HA and interacts with heavy chains of IαI proteins, is another protein essential for the formation and stability of expanded HA-rich oocyte- cumulus ECM in mice and pigs [18,23,26]. Together with the high sequence similarity found among human, murine, rat, equine and porcine TNFAIP6 [23,25,27,28] and the expression of this protein and/or the respective gene in ovarian follicles of all of the examined species [18,23,25,26,27,28,43], it strongly supports the concept that TNFAIP6-mediated covalent binding of HCs (of IαI proteins) onto HA is a mechanism that mammalian OCC have in common [22].

### 2.3. PTX3

Experiments performed with *Ptx3* knockout mice [29] have shown that complexes from *Ptx3*−/−mice have defective cumulus matrix organization. Interestingly, hormone stimulation of *Ptx3*−/−OCC in vitro showed that while cumulus cells synthesized HA at a normal rate they were unable to organize this polymer in the cumulus ECM [30]. PTX3 is essential protein for organizing the HA polymer in the cumulus ECM in mice [30,31,32]. PTX3 protein plays role in cumulus ECM assembly, where HCs transferred from IαI to HA by the catalytic activity of TNFAIP6 bind distinct protomers of multimeric PTX3 [33]. In pigs, *PTX3* transcripts were significantly increased in OCC 24 h after in vivo hCG or in vitro FSH/LH stimulation [34]. Western blot analysis with PTX3 antibody revealed that cumulus ECM extracts from both in vivo hCG-stimulated pigs and in vitro FSH/LH-stimulated OCC cultured in medium supplemented either with follicular fluid or porcine serum, contain high levels of PTX3 protein. The localization of PTX3 protein in the porcine OCC was confirmed by immunostaining [34]. The mouse data concerning the integrity of HA-rich oocyte-cumulus ECM [30,31] together with porcine data [34] demonstrated the importance of PTX3 protein in the ovarian follicles.

## 3. Effect of Specific Inhibitors (AG1478, Lapatinib, Indomethacin and MG132) and Progesterone Receptor Antagonist (RU486) on the Formation of HA-Rich Cumulus Extracellular Matrix

### 3.1. Inhibition of EGFR Signaling Pathway (with AG1478) Affects Meiotic Maturation, Cumulus Expansion and Hyaluronan and Progesterone Synthesis

Several observations support the finding that EGF-like growth factors, i.e., amphiregulin and epiregulin, produced by granulosa cells and cumulus cells play a major role in triggering oocyte maturation and the cumulus expansion of OCC in mice [44]. Epidermal growth factor (EGF) is a poor inducer of porcine cumulus expansion in vitro [45]. Nevertheless, FSH pre-treatment strongly enhances EGF response within 3 h, as evidenced by a high increase in HA production and cumulus expansion after sequential exposure to FSH and EGF [46]. FSH itself does not affect epidermal growth factor receptor (EGFR) concentration or the tyrosine phosphorylation of EGFR, but it enhances the EGF-induced tyrosine phosphorylation of EGFR, indicating that the FSH signaling pathways may stimulate or modulate specific EGFR–regulating proteins. FSH also rapidly induces porcine OCC to express EGF-like growth factors [47] and TACE/ADAM17, a protease that cleaves and activates the EGF transmembrane precursors [48]. It has been shown [45] that AG1478, the inhibitor of EGFR tyrosine kinase activity, reduces 50% of the synthesis and 90% of the HA retained in the cumulus ECM, and prevents the expansion of porcine OCC stimulated with FSH for 24 h in vitro culture. Furthermore, although EGF does not stimulate progesterone production by porcine OCC and granulosa cells, the pre-treatment of both cell types with inhibitor AG1478, significantly reduces the stimulatory effect of FSH on progesterone production. This result is in agreement with the previous finding showing that AG1478 reduced the FSH-induced expression of the steroidogenic enzyme P450 side chain cleavage, *Cyp11a1*, in rat granulosa cells [49]. Importantly, the addition of AG1478 to the culture medium, irrespective of the stimulation, inhibited nuclear maturation in pigs [45,47]. Similarly, Ashkenazi et al. [50] observed that after the local administration of AG1478 inhibitor into the rat ovary, the ratio of entrapped immature oocytes (in germinal vesicle stage) in the inhibitor-treated ovaries was 5-fold higher than in the contralateral untreated ovaries. Thus, results in pigs [45] showing that EGFR activation by EGF-like growth factors produced under the FSH stimulus is involved in initiating the ovulatory events in porcine OCC are consistent with the results obtained in mice and rats [44,50]. However, it is possible that FSH *trans*-activates EGFR via mechanisms independent of EGF shedding [48]. Finally, it is important to note that the FSH-induced synthesis of both HA and progesterone is reduced but not abolished by AG1478, indicating that other signaling pathways elicited by FSH are operating in parallel [45].

### 3.2. Inhibition of EGFR Tyrosine Kinase (with Lapatinib) Affects Meiotic Maturation, Cumulus Expansion, and Expression of Cumulus-Associated Transcripts

In the ovarian follicles, EGFR mediates the ovulatory response to LH and the sustained activity of EGFR is an absolute requirement for LH-induced oocyte maturation and cumulus expansion [51]. However, abnormally elevated EGFR kinase activity can lead to various pathological states, including cancer. The human epidermal growth factor receptor (HER) family consists of four closely related transmembrane receptors: HER1 (human epidermal growth factor receptor 1, EGFR), HER2/c-Erb-B2, HER3/Erb-B3 and HER4/Erb-B4. These members of the type I receptor tyrosine kinase family are frequently implicated in cancer [52,53]. HER family-related downstream signaling plays a crucial role in cell proliferation, survival, migration and differentiation [54,55]. Recently, it has been investigated the effect of lapatinib on processes essential for ovulation, such as oocyte meiotic maturation and cumulus expansion, since it has been demonstrated that using of biological agents for treating cancer in women increases the probability that some women will conceive while taking the inhibitor (lapatinib) of growth factor signaling [56]. Lapatinib (GW572016, Tykerb/Tyverb; GlaxoSmithKline) is an orally active small molecule that reversibly and selectively inhibits the tyrosine kinase domain of both EGFR and HER2 [57] by binding to the ATP-binding site of the kinase, and preventing autophosphorylation or the rapid development of resistance to monotherapies [58]. It has been found that lapatinib, through the EGFR signaling pathway, is able to inhibit oocyte maturation in pigs [59]. In addition, lapatinib is able to reduce the expression of cumulus expansion -related transcripts (*TNFAIP6*, *PTGS2*), HA synthesis, cumulus expansion and progesterone secretion by porcine OCC cultured in FSH/LH supplemented medium [59]. This is in good agreement with the previous study showing the reduction of FSH-induced synthesis of both HA and progesterone by AG1478, another inhibitor of EGFR tyrosine kinase activity [45].

### 3.3. Addition of Progesterone Receptor Antagonist (RU486) to Culture Medium Affects Meiotic Maturation; It Does Not Affect Formation of Cumulus Extracellular Matrix Relying on the Covalent Transfer of Heavy Chains of IαI Molecules to Hyaluronan

Progesterone is an ovarian steroid hormone that regulates key aspects of female reproduction and acts through the progesterone receptor (PR). The progesterone receptor is a member of the nuclear receptor superfamily and functions as a ligand-activated transcription factor. The functional roles of PR in the ovary have been investigated with genetically modified mouse models and PR antagonist (RU486). It has been demonstrated that PR-knockout mice do not ovulate and are infertile [60,61]. Interestingly, despite the failure of ovulation in PR-null mice, cumulus expansion proceeds normally. However, the addition of RU486 to the culture medium with porcine OCC significantly decreases FSH/LH-induced resumption of oocyte meiosis (~74%; *p* < 0.05) and progression of oocyte maturation to the MII stage (~44%; *p* < 0.05) [62]. Gonadotropins stimulate cumulus expansion as well as HA synthesis by porcine OCC during in vitro maturation [19]. The addition of RU486 did not change FSH/LH-stimulated total HA synthesis; however, the retained amount of HA within the complexes was significantly reduced (*p* < 0.05). The amount of HA retained in cumulus ECM was approximately 60% of the amount retained within the cumulus ECM of the OCC cultured with FSH/LH alone [62]. However, the immunodetection of HABP, TNFAIP6, and PTX3 proteins in FSH/LH-stimulated OCC treated with RU486 confirmed the spatial localization of cumulus-associated components [62]. Furthermore, western blot analysis detected the heavy chains of IαI proteins in the matrix extracts of FSH/LH stimulated-OCC, treated with RU486 [62]. Shimada et al. found [63] that porcine OCC cultured in vitro in the presence of FSH/LH and RU486 had little developmental competence to proceed to the blastocyst stage. Moreover, RU486 significantly impaired blastocyst development in mice [64] and in cows [65]. Also, the administration of RU486 by intraperitoneal injection to gonadotropin-primed mice reduced the number of ovulated oocytes [66] and in mouse follicles cultured with hCG/EGF, in the presence of RU486, the MII rate was significantly lower (62%) [67]. Surprisingly the addition of RU486 to the culture medium significantly increased progesterone production by porcine OCC compared to FSH/LH alone [62]. To summarize, in pigs, the inhibition of PR with RU486 does not affect HA synthesis, the formation of cumulus ECM and covalent linkage between HA and heavy chains of IαI, but it appears that progesterone may be critical for maintaining an optimal microenvironment for oocyte maturation and fertilization [62].

### 3.4. Addition of General COX Inhibitor (Indomethacin) to Culture Medium does not Affect Meiotic Maturation, nor Formation of Cumulus Extracellular Matrix

In mammalian species, the preovulatory surge in gonadotropins upregulates the follicular expression of cyclooxygenase-2 (COX-2), which elevates the levels of prostaglandins [68,69]. Mice genetically deficient in *Cox-2* exhibited ovulatory failure [70,71]. In addition, *Cox-2* and prostaglandin E2 receptor subtype *Ep2* null mice were infertile [72]. The administration of either general COX inhibitors (indomethacin) or inhibitors selective for COX-2 (NS-398, celecoxib) reduced ovulation rates in rodents, domestic animals, and monkeys [73,74,75]. In porcine OCC cultured in FSH/LH supplemented medium for 44 h, neither the resumption of meiosis (~87%) nor progression of oocyte maturation to MII (~72%) was affected by indomethacin [62]. Concomitantly, the total HA synthesis and retained amount of HA within the complexes was similar to those of OCC stimulated with FH/LH alone. In addition, the covalent binding between heavy chains of IαI molecules to HA in the cumulus ECM extracts, as well as cumulus ECM-related proteins (HABP, TNFAIP6, and PTX3) were detected in FSH/LH-stimulated OCC treated with indomethacin [62]. In agreement, indomethacin did not block HA synthesis induced by FSH in Graafian follicles in mice [76]. Moreover, Western blot analysis confirmed that in cumulus cells of *Cox-2* and *Ep2* null mice, the TNFAIP6 protein remained covalently associated with the heavy chains of IαI molecules. This is clear evidence that the ovaries of *Cox-2* null mice maintain the capacity to produce *Has2* mRNA in response to an ovulatory dose of hCG as well as the ability to form expanded cumulus ECM [72]. Matsumoto et al. [77] showed that the ovulatory process, but not follicular growth, oocyte maturation or fertilization, was primarily affected in adult *Cox-2* or *Ep2*-deficient mice. Eppig [76] suggested that PGE2 might play a role in the indirect stimulation of cumulus expansion by LH. Interestingly, Ben-Ami et al. [78] have shown that LH may mediate its effects on COX-2 expression in cumulus cells via the induction of the EGF-related factors amphiregulin, epiregulin, and betacelulin produced in human granulosa cells. Similarly, in mice, these factors bind EGF receptor in cumulus cells and induce the *Cox-2* message [79]. Also Hsieh et al. [80] have demonstrated that in mice, the LH-induction of *Cox-2*/*Ptgs2* expression is dependent on the activation of EGF receptor signaling in cumulus and mural granulosa cells. Moreover, it has been shown that lapatinib, the inhibitor of EGFR tyrosine kinase activity, reduces the expression of *COX-2* mRNA in porcine OCC cultured in vitro [59]. To summarize, in pigs, the inhibition of COX-2 by indomethacin does not affect FSH/LH-stimulated HA synthesis and the formation of the covalent linkage between heavy chains of IαI to HA nor progesterone production by cultured OCC [62].

### 3.5. Inhibition of Proteasomal Proteolysis (with MG132) Strongly Affects Meiotic Maturation and Formation of Cumulus Extracellular Matrix 

Protein turnover mediated by the ubiquitin-proteasome pathway plays an essential role in cell physiology and pathology. Ubiquitin is a small chaperone protein that forms covalently linked isopeptide chains on protein substrates to mark them for degradation by the 26S proteasome. The 26S proteasome is a multicatalytic protease complex that specifically recognizes and hydrolyzes proteins tagged with multiubiquitin chains. The subunits of the 26S proteasome comprise approximately 1% of the total proteome in mammalian cells; the ubiquitin-proteasome pathway serves as the main substrate-specific cellular protein degradation pathway [81,82,83]. MG132 is a cell-permeable peptide aldehyde that inhibits the chymotrypsin-like activity of the 20S proteasomal core [84,85]. The ubiquitin–proteasome pathway modulates mouse oocyte meiotic maturation and fertilization via regulation of the MAPK-cascade and cyclin B1 degradation [86,87]. In addition, the role of the 26S proteasome in the regulation of oocyte meiosis has been described in mammals, specifically rats, mice and pigs [83,88,89,90]. In pigs, the addition of MG132 to the gonadotropin-supplemented medium prevented cumulus expansion and significantly reduced HA synthesis by the cumulus cells. Moreover, the covalent binding between HA and heavy chains of IαI was not detected in the MG132-treated porcine OCC [89]. The formation of expanded HA-rich cumulus ECM depends on HA association with specific hyaluronan-binding proteins [41], such as IαI [12,20], TNFAIP6 [23,24,25,26,27] and PTX3 [30,34], which all have been detected in mice and pigs. The TNFAIP6-HC complex is likely a catalyst in the transfer of heavy chains (of IαI) onto HA [91]. This mediator reacts rapidly with any HA, leading to the formation of heavy chain-HA and release of the TNFAIP6 catalyst. While the mRNA expression of *HAS2* and *TNFAIP6* in the gonadotropin-stimulated OCC was increased in pigs [27], mice [17,23], and rats [25], in the presence of MG132, the expression of *HAS2* and *TNFAIP6* was markedly suppressed in porcine OCC. In addition no signal of HA was observed by immunostaining in porcine OCC [92]. Tsafriri et al. [93] used a broad-spectrum metalloprotease inhibitor, GM6001, to find whether proteolytic activity was involved in the action of LH on the resumption of meiosis in rats. His conclusion was that this inhibitor prevented the LH-induced resumption of meiosis. In agreement, the inhibition of proteasomal proteolysis with MG132 arrested 90% of porcine oocytes in the germinal vesicle stage. Moreover, MG132 blocked the degradation of F-actin-rich transzonal projections (TZPs) interconnecting cumulus cells with the oocyte and cumulus expansion in pigs [89]. The resumption of oocyte meiosis was accompanied by the disappearance of the zona pellucida-spanning and actin microfilament-rich TZPs, and an alteration of gap junction communication [16,89,94]. Since the maintenance of TZPs supports an oocyte meiotic block and porcine OCC treated with MG132 remain unexpanded, it has been suggested that proteasomal proteolysis participates in the process of the resumption of meiosis. The terminal differentiation of cumulus oophorus within the ovarian follicle plays a crucial role in the ability of the oocyte to resume meiosis and reach full developmental competence [95,96,97]. Progesterone has been shown to enhance the activity of proteolytic enzymes important for the rupture of the follicular wall at ovulation [98]. Gonadotropins induce PR expression in cumulus cells concomitantly with an increase in progesterone secretion by porcine OCC [99,100]. The involvement of the proteasome in the turnover of StAR has been described [101,102,103] with subsequent influence on progesterone synthesis [102]. The transfer of cholesterol across the mitochondrial membranes is promoted by StAR [104]. In MG132-treated porcine OCC the progesterone levels were reduced [92]. In contrast, Tajima et al. [102] found a significant elevation in progesterone synthesis in MG132-treated rat granulosa cells. This discrepancy can be explained by the differences in the cell culture regimen. The relation between progesterone and proteolytic enzyme activity during ovulation in the gonadotropin-treated immature rat ovary was studied by Iwamasa et al. [98]. Their results suggested that progesterone played an indispensable role during the first 4 h of the ovulatory process by regulating proteolytic enzyme activities. Our results showed that the ability of gonadotropin-stimulated porcine cumulus cells to produce progesterone to a level comparable with control OCC was not restored when MG132 was present for 20 h of the culture, but it was restored (50%) when MG132 was only present for 3 h. In summary, the specific proteasomal inhibitor MG132 prevents the gonadotropin-induced resumption of meiosis and subsequent cumulus expansion. In addition, it protects TZPs against breakdown, affects the terminal differentiation of cumulus cells, markedly reduces the expression of *HAS2* and *TNFAIP6* and prevents the formation of a covalent linkage between HA and the heavy chains of IαI [89,92].

## 4. Conclusions

Ovulation is controlled through multiple inputs including endocrine hormones, immune and metabolic signals, as well as intra-follicular paracrine factors from the theca, mural and cumulus granulosa cells and the oocyte itself. The ovulatory mediators exert their effects through remodeling of the cumulus ECM that surrounds the oocyte. The proper structure of the cumulus ECM, which is essential for ovulation, transport of the OCC to the oviduct and fertilization depends on the interaction between HA and HA-associated ECM proteins. HA cross-linking within the cumulus ECM represents an important new mechanism in the regulation of the ovulatory process in mammalian follicles. We suggest that the structural changes in cumulus ECM affect signaling pathways and consequently the resumption of meiosis. In addition, it is interesting to note that the synthesis of ECM molecules is controlled by specific growth factors and the life of ECM molecules is determined by proteases. Thus, growth factors signaling pathways and the control of ECM turnover by proteases become possible targets for new therapies.

## Figures and Tables

**Table 1:** Gonadotropin-induced matrix components essential for the formation and stability of the HA-rich oocyte-cumulus ECM.

ECM Component	Description	Tissue Location (Oocyte-Cumulus Complexes-OCC)	Species	References
HA	Hyaluronan	OCC	Mice	[15,16,17,18]
		OCC	Pigs	[19]
IαI	Inter-alpha-trypsin inhibitor (also called inter-alpha-trypsin inhibitor (ITI))	OCC	Mice	[10,18,20,21]
		OCC	Pigs	[12,22]
TNFAIP6	Tumor necrosis factor alpha-induced protein 6 (also called Tumor necrosis factor stimulated gene-6 (TSG-6))	OCC	Mice	[13,18,23,24]
		Ovary	Rats	[25]
		OCC	Pigs	[26,27]
		Ovarian follicles	Equine	[28]
PTX3	Pentraxin 3	OCC	Mice	[29,30,31,32,33]
		OCC	Pigs	[34]

## Abbreviations

ECM	Extracellular matrix
HA	Hyaluronan
IαI	Inter-alpha-trypsin inhibitor
TNFAIP6	Tumor necrosis factor alpha-induced protein 6
PTX3	Pentraxin 3
OCC	Oocyte-cumulus complexes
COX2/PTGS2	Cyclooxygenase/prostaglandin endoperoxide synthase
EGF	Epidermal growth factor
EGFR	Epidermal growth factor receptor
GVBD	Germinal vesicle breakdown
M I	Metaphase I
M II	Metaphase II
hCG	Human chorion gonadotropin
PMSG	Pregnant mare gonadotropin
TFGβ	Transforming growth factor beta
